# Patient journey and treatment pattern in myasthenia gravis: real-world data from the Brazilian public health system

**DOI:** 10.1055/s-0045-1813233

**Published:** 2026-01-25

**Authors:** Rosana Herminia Scola

**Affiliations:** 1 Universidade Federal do Paraná, Departamento de Medicina Interna, Disciplina de Neurologia, Curitiba PR, Brazil.

The study *“Patient Journey and Treatment Pattern in Myasthenia Gravis: Real-World Data from the Brazilian Public Health System”* 1 represents an important step toward understanding myasthenia gravis (MG) within Brazil's public healthcare network. Drawing on data from more than 13,000 patients treated through the Brazilian Unified Health System (Sistema Único de Saúde – SUS) between 2010 and 2023, the authors provide an unprecedented overview of the clinical characteristics, management strategies, and healthcare utilization patterns of this patient population.

The steady increase in MG admissions and outpatient encounters mirrors the global rise in disease prevalence. 2 3 4 This likely reflects better recognition, improved diagnostic tools, and longer patient survival, but it also exposes the mounting pressure MG places on healthcare systems. The authors report that nearly one-third of patients experienced exacerbations, and about 10% required hospitalization for myasthenic crisis—a reminder that disease control remains elusive for many. The increase in case fatality, from 0.76% in 2011 to 1.9% in 2023, underscores the need for stronger preventive strategies and earlier intervention. 5 6 These data align with recent population-based studies showing that, despite therapeutic progress, mortality from MG continues to vary widely across regions and healthcare contexts.

The treatment patterns described reflect both the limitations and the realities of MG management within the SUS. Azathioprine and pyridostigmine remain the cornerstones of therapy, but frequent dose changes, treatment switches, and prolonged dependence on intravenous immunoglobulin (IVIg) suggest persistent difficulty achieving symptom stability. Such reliance on older agents likely stems from restricted access to newer immunotherapies and delays in treatment escalation. 7 8 Similar patterns have been reported internationally, where a subset of patients continues to experience refractory disease despite standard care. 9 The use of IVIg as an initial or chronic therapy further emphasizes the severity of illness in this cohort and the lack of sustainable disease control in some cases. 10 11

These findings call attention to the urgent need to modernize MG treatment in Brazil's public sector. Equally important are systemic strategies such as structured referral pathways, standardized monitoring protocols, and early identification of refractory cases. Together, these measures could reduce hospitalizations, improve quality of life, and lessen the economic burden of care.

This study highlights the magnitude and challenges in the diagnosis and treatment of myasthenia gravis (MG) within the SUS. However, due to the characteristics of the SUS database, essential diagnostic information is lacking, such as serum antibody data (anti-AChR, anti-MuSK, anti-titin, and anti-LRP4), as well as neurophysiological studies. 12

Regarding therapy, the data are incomplete, as there is no record of corticosteroid use—the main treatment following pyridostigmine. 7 Information on thymectomy is also absent, despite its established role in the management of selected patients, as demonstrated in the MGTX trial. 13

These findings reinforce the urgent need to update the current SUS treatment protocol (LOTS), which remains outdated and does not adequately include corticosteroid therapy, as illustrated in Table 1 of the article.

In essence, the study fills a long-standing knowledge gap in Brazilian MG research. It also serves as a reminder that the benefits of modern therapies must reach patients equitably across different healthcare settings.
